# Impact of Probiotic Supplementation and High-Intensity Interval Training on Primary Dysmenorrhea: A Double-Blind, Randomized Controlled Trial Investigating Inflammation and Hormonal Modulation

**DOI:** 10.3390/nu17040622

**Published:** 2025-02-09

**Authors:** Min-Yi Yang, Hao-Yu Chen, Chi-Hong Ho, Wen-Ching Huang

**Affiliations:** 1 Department of Exercise and Health Science, National Taipei University of Nursing and Health Sciences, No. 365, Ming-te Road, Peitou District, Taipei City 112303, Taiwan; m331518@gmail.com (M.-Y.Y.); a0922319460@gmail.com (H.-Y.C.); 2 Department of Obstetrics and Gynecology, Taipei Veterans General Hospital, Taipei City 11217, Taiwan; hochwater@gmail.com

**Keywords:** primary dysmenorrhea, probiotics, inflammation, menstrual distress, hormones, health promotion

## Abstract

**Background**: Dysmenorrhea, categorized as primary (PD) or secondary (SD), significantly affects females during their reproductive years, impairing quality of life, performance, and social relationships. Alongside medical treatment, exercise and probiotics are complementary measures for managing PD and promoting health. This study examined the impact of probiotic supplementation and high-intensity interval training (HIIT) on PD severity, physiological modulation, and physical fitness. **Methods**: Participants, recruited according to the primary dysmenorrhea criteria, were divided into non-PD (control) and PD groups, with the PD group further classified into dysmenorrhea (Dysmen), dysmenorrhea with probiotics (DysmenPro), dysmenorrhea with exercise (DysmenEx), and dysmenorrhea with both (DysmenExPro). Interventions included 10 weeks of HIIT and probiotics. Pre- and post-intervention assessments included questionnaires on premenstrual and menstrual symptoms, physical fitness evaluations, and blood sample analyses for biochemical, hormonal, and prostaglandin levels. **Results**: HIIT significantly reduced premenstrual symptoms, menstrual distress, and pain severity, likely due to hormone (estradiol, prolactin, progesterone, cortisol) modulation and decreased inflammation (high-sensitivity *C*-reactive protein, PGE2, PGF2α). Cardiovascular endurance and explosive strength showed improvement through high-intensity interval training (HIIT), whereas probiotics had no significant effect on these aspects of physical fitness. While probiotics reduced premenstrual and menstrual distress symptoms, they had no notable impact on pain, inflammation, or hormone levels. Dysmenorrhea-related discomforts were correlated significantly with inflammation and hormones. **Conclusions**: The intervention strategy involving probiotics and HIIT exercise may be utilized as an alternative and complementary treatment to alleviate PD symptoms. Furthermore, this strategy could also be incorporated into educational health plans to promote women’s health and potentially prevent gynecological disorders in the adolescent population.

## 1. Introduction

The menstrual cycle is defined from the first day of menstruation to the first day of the next cycle. It is composed of four phases (menstruation, the follicular phase, ovulation, and the luteal phase) and typically lasts between 23 and 35 days. The reproduction system is strictly regulated by the hypothalamic–pituitary–gonadal axis through the gonadotropin-releasing hormone, which is released from the hypothalamus to the secretory cells of the adenohypophysis in the pituitary. This mechanism results in the release of follicular-stimulating hormone (FSH) and luteinizing hormone (LH) into the circulation system, which perform gonadal functions. Gonadotropins, specifically FSH and LH, primarily target granulosa and theca cells in the ovaries. These hormones facilitate the expression of estrogen receptors and the production of steroid hormones, such as androgens, estrogens, and inhibins. Through mechanisms of positive and negative feedback, they regulate the menstrual cycle and support ovarian maturation [1]. Menarche is characterized as the last event of puberty (age: 8–13 years); the monthly menstrual cycle consecutively occurs between menarche (age: approximately 11–15 years) and menopause (age: approximately 45–55 years), representing the reproductive life of a person. The pathogenic mechanism underlying dysmenorrhea could be the increased secretion of prostaglandin F2α (PGF2α) and prostaglandin E2 (PGE2), leading to myometrial contractions, vasoconstriction, uterine ischemia, and pain [2]. Dysmenorrhea is a common primary gynecological disease with a prevalence of 90% among college students; it has been shown that dysmenorrhea has a great impact on academic performance [3]. Dysmenorrhea is clinically categorized as either primary dysmenorrhea or secondary dysmenorrhea, with pathological syndromes, including endometriosis, adenomyosis, and uterine fibroids, as well as pelvic anatomic abnormalities in SD being the main differences between SD and PD [4].

Dysmenorrhea is diagnosed by physical examination and anamnesis for PD, while ultrasound examination is often used to exclude SD. The clinical treatment of dysmenorrhea can be conducted through medical treatments (non-steroidal anti-inflammatory drugs and hormone therapy) or surgical treatment, although surgical treatment is often reserved for patients of SD that experience medical treatment failure, infertility, or symptomatic lesions [5]. Regarding nutritional strategies for dysmenorrhea, a quantitative meta-analysis of randomized clinical trials revealed that the degree of pain could be significantly decreased with vitamin D supplementation [6]. Another systematic review highlighted that physiotherapy interventions for PD led to a clinically significant reduction in menstrual pain intensity. Females may consider this option alone or in combination with other therapeutic modalities [7]. From the perspective of complementary and alternative medicine, acupuncture therapy and manipulation also showed the significant alleviation of pain intensity and PD syndromes. Moreover, herbal prescriptions and Chinese herbal medical enemas may be effective in relieving dysmenorrhea and are associated with fewer side effects compared with pharmacological treatments [8,9]. A meta-analysis of low-quality evidence demonstrated that exercise led to a clinically significant improvement in menstrual pain intensity (25 mm based on the visual analog scale [VAS]). These findings suggested the use of exercise in conjunction with other modalities for menstrual pain management [10]. The appropriate exercise protocols and combined therapeutic modalities should be further investigated for the management of gynecological diseases.

High-intensity interval training (HIIT) is a popular exercise model. According to a survey conducted by The American College of Sports Medicine’s Health and Fitness Trends, it ranked 7th and 20th in popularity in 2023 and 2024, respectively. HIIT (i.e., heart rate-based training) is composed of repeated short bouts, requires near-maximum effort exercise (>80% maximum heart rate), and is followed by active or passive recovery periods as multiple-interval exercise training. Based on this concept, HIIT workouts can be programmed using various equipment options (e.g., treadmills, rowers, training rope, or bikes) to achieve cardiometabolic health benefits, such as peak aerobic capacity, body composition, and glucose and lipid metabolism [11]. In addition, a systematic review showed that HIIT exercise could enhance mitochondrial-associated adaptation in overweight and obese individuals through the regulation of oxidative stress and inflammation [12]. In chronic inflammation disease, the high level of expression of proinflammatory biomarkers (*C*-reactive protein, tumor necrosis factor-α, and cell adhesion molecules) in patients with chronic heart failure could be decreased through the implementation of high-intensity aerobic interval training [13]. Furthermore, HIIT mitigated the tissue lesion, hypercoagulable state, and systemic inflammation in a surgically induced endometriosis animal model [14].

The World Health Organization (WHO) defines probiotics as “live microorganisms that provide health benefits to the host when administered in adequate amounts” [15]. Functional probiotics exerted multiple physiological benefits for health promotion and disease alleviation through their metabolites and the modulation of the gut microbiota [16]. The role of probiotics in mental health has been supported by research demonstrating their ability to ameliorate depression and anxiety through neuroactive substances in the gut–brain axis, such as gamma-aminobutyric acid and serotonin. Their potential lies in their ability to reduce inflammation and dampen the activation of the hypothalamic–pituitary–adrenal axis, thereby alleviating symptoms of depression and anxiety [17]. In our previous studies, *Lactobacillus plantarum* PS128 improved depressive symptoms by ameliorating inflammatory responses and regulating the levels of cortisol, dopamine, and serotonin [18]. Furthermore, it was shown that the supplementation of PS128 during systemic inflammation induced by high-intensity endurance training improved oxidative stress, inflammation, and exercise-induced fatigue by modulating gut microbiota and their metabolites [19]. The muscular injury caused by endurance exercise training could be significantly decreased by the supplementation of *Bifidobacterium longum* OLP-01 [20]. In a previous study, the supplementation of patients with dysmenorrhea with a probiotic mixture led to improvement in mental health and a reduction in the use of analgesics; nevertheless, there was no significant change in inflammatory cytokines [21]. However, studies assessing the potential effects of probiotic supplementation on dysmenorrhea are limited. Based on our previous evidence regarding the effectiveness of HIIT on dysmenorrhea [22], the supplementation of probiotics could be further considered as part of health management in women.

In the present study, eligible female participants were randomly assigned to treatment groups and subjected to double-blind probiotic supplementation to investigate the potential effects of probiotic and high-intensity interval training interventions on primary dysmenorrhea. The physiological modulation and comprehensive outcomes (Premenstrual Symptoms Screening Tool [PSST], Menstrual Distress Questionnaire [MDQ], Short-Form McGill Pain Questionnaire [SF-MPQ], biochemical variables, sex hormones, and prostaglandins) were further investigated. The present study sought to evaluate the effects of probiotics, both alone and in combination with exercise, on the synergistic alleviation of primary dysmenorrhea discomfort and distress symptoms, as well as their impact on inflammation, hormone regulation, and improvements in physical fitness.

## 2. Materials and Methods

### 2.1. Experimental Design

**Figure 1:**
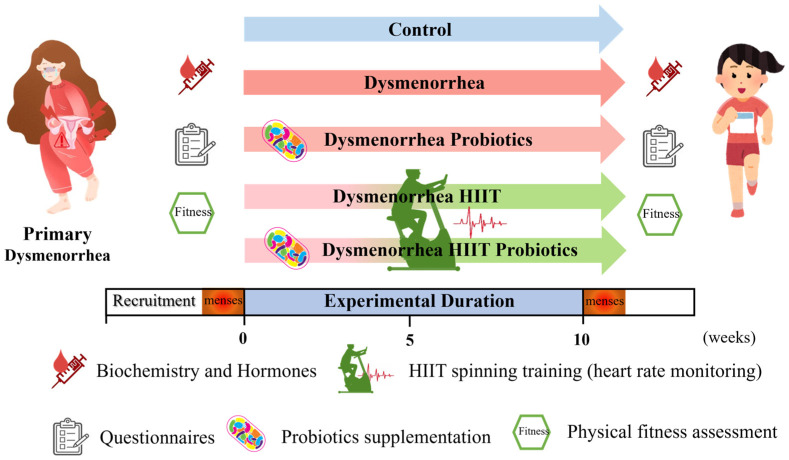
Experimental scheme: The participants with PD were randomly allocated to the dysmenorrhea with placebo (Dysmen), dysmenorrhea with probiotics (DysmenPro), dysmenorrhea with HIIT (DysmenEx), and dysmenorrhea with HIIT and probiotics (DysmenExPro) groups. HIIT intervention and probiotic supplementation were conducted over a 10-week period. During the HIIT sessions, participants were required to reach a heart rate of at least 85% of their maximum. Blood samples were collected approximately 3 days prior to the menstrual cycle to evaluate biochemical variables, hormone levels, and prostaglandin levels. Additionally, before and after the HIIT intervention, participants completed the associated questionnaires and underwent physical fitness assessments.

**Figure 2:**
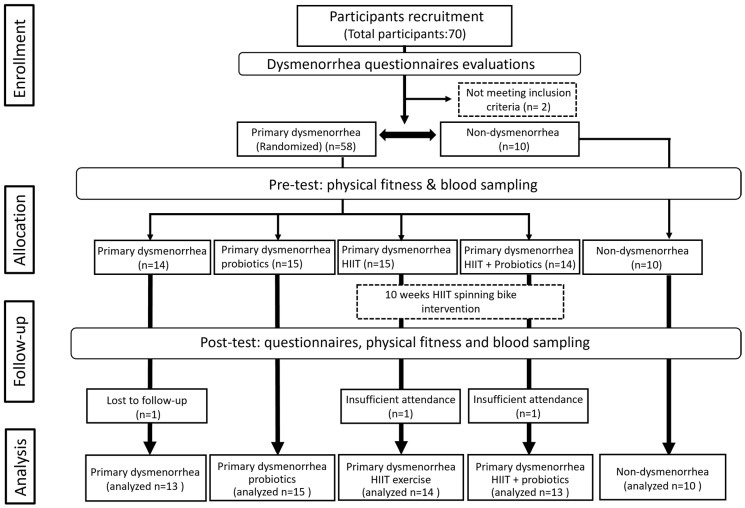
Study flow diagram: This study was a randomized, parallel-controlled clinical trial. Prior to and following the experimental intervention, participants completed questionnaires and underwent physical fitness assessments. Additionally, blood samples were collected three days before menstruation, as well as before and after the intervention, to evaluate biochemical variables and hormone levels associated with menstrual pain.

This study was designed as a randomized double-blind trial. Participants in the control (n = 10; non-PD) and PD (n = 58) groups were selected based on the inclusion criteria stated below. The PD group was randomly divided into PD with placebo (Dysmen; n = 14), PD with HIIT and placebo (DysmenEx; n = 15), PD with probiotics (DysmenPro; n = 15), and PD with HIIT and probiotics (DysmenExPro; n = 14) groups. Blood sampling, questionnaire assessments (including the PSST, MDQ, and SF-MPQ) and evaluations of participants’ physical fitness were conducted both before and after the experimental intervention. Additionally, blood samples were collected approximately three days prior to the onset of the menstrual cycle to analyze biochemical variables and hormone levels. The 10-week intervention of exercise training and probiotic supplements was implemented in the groups indicated. All subjects provided informed consent prior to their participation in the exercise program, probiotic supplementation, and related assessments. The experimental scheme of the study and the flow diagram illustrating the study design are presented in Figure 1 and Figure 2, respectively.

### 2.2. Subjects

The inclusion criteria for this study were as follows: participants were aged 18–40 years, the presence of primary dysmenorrhea (PD) was required, and specific severity levels, determined based on the visual analogue scale (VAS) score of the SF-MPQ (0–2 for the non-dysmenorrhea control group; >5 for the dysmenorrhea group), were mandated. Exclusion criteria were described in a previous study [22]. The participants were female students recruited from our faculty. Five subjects were excluded or dropped out from the study because they did not meet the inclusion criteria, had an insufficient attendance rate (<80%), or were lost to follow-up. As a result, 65 participants completed all experimental procedures required for data analysis. Participants were also instructed to refrain from using nutrient supplements and to maintain their usual lifestyle throughout the duration of the study. Participant characteristics related to anthropometric and menstrual data are presented in Table 1.

### 2.3. Probiotics

Probiotic supplementation was designed as a double-blind experiment within non-exercise (Dysmen and DysmenPro) and exercise (DysmenEx and DysmenExPro) training groups. The probiotic mixture, including *Bifidobacterium longum* subsp. longum OLP-01 (OLP-01), *L. plantarum* PL-02 (PL-02), and *Lactococcus lactis* LY-66, (ratio 1:1:1), was prepared in capsules by Glac Biotech Co., Ltd. (Tainan, Taiwan). Each capsule included 300 mg of lyophilized probiotics powder, equivalent to 1.5 × 10^10^ colony-forming units, and 100 mg of excipient of microcrystalline cellulose. Placebo capsules contained 400 mg of excipient of microcrystalline cellulose. The probiotics and placebo capsules were administered once per day (one capsule/time) after a meal. The probiotics used in this study—OLP-01, PL-02, and LY-66—were originally isolated from a female Olympic weightlifting champion [23]. Our previous studies have demonstrated that the probiotic strain OLP-01 can mitigate inflammation [24] and oxidative stress [20], contributing to enhanced endurance performance. Additionally, supplementation with PL-02 and LY-66, when combined with exercise training, has been shown to enhance physical fitness, body composition, and gastrointestinal health by modulating the gut microbiota [25]. These probiotics, either individually or in combination with HIIT, were utilized in the current study to evaluate their effects on PD alleviation.

### 2.4. HIIT Intervention

The group class, led by a certified cycling coach, was implemented with HIIT. Before the program, participants received instructions on the safe and proper use of spinning bikes. They attended two sessions weekly for 10 weeks, with a minimum 80% attendance requirement. Sessions included a 5 min warm-up, 15–20 min of HIIT (15 sprints of 20 s with 40 s active recovery), a 5 min cool-down, and stretching. Intensity was adjusted using the Borg scale and heart rate belt (Obeat1, Alatech, Taichung, Taiwan), with a consistent cycling pace and out-of-saddle (standing position) sprints during high-intensity (all-out) phases. Sessions were adapted slightly based on prior research [22], ensuring recovery days between classes for optimal performance and safety. From the 7th week onward, the duration of the all-out phase was increased to 25 s, while the rest of the intervals remained unchanged.

### 2.5. Menstrual Questionnaire Surveys

The PSST was used to evaluate the severity of premenstrual syndrome symptoms and their impact on participants’ daily activities [26]. The MDQ, originally developed by Moos et al. in 1968, was composed of eight dimensions (pain, water retention, autonomic reactions, negative effects, impaired concentration, behavioral changes, arousal, and control) [27]. A modified MDQ, validated for internal consistency (Cronbach’s α = 0.83) [28], was used for related syndromes and discomforts during both the premenstrual and menstrual phases. The SF-MPQ, a widely used tool for quantifying the quality and intensity of pain, includes sensory and affective variables. The correlation between the results of the standard MPQ and the SF-MPQ was consistently high and statistically significant [29]. In addition, the VAS, represented by a 10 cm rating scale, was used to rank the overall degree of pain (dysmenorrhea in the present study).

### 2.6. Hormone, Prostaglandins, and Biochemical Analyses

Participants were asked to record the start of their menstrual period to ensure that blood samples were collected approximately three days prior to menstruation. A clinical research nurse drew 10 mL of whole blood from the median cubital vein on the forearm into tubes containing clot activators and K2-ethylenediamine tetraacetic acid. After centrifugation at 3000× *g* for 10 min, the serum and plasma were separated and stored immediately at −20 °C for further biochemical and hormone analysis. The clinical biochemical variables and hormones, as described in a previous study [22], were measured using the AU5800 Clinical Chemistry Analyzer (Beckman Coulter, Tokyo, Japan) and the UniCel DxI 800 Access Immunoassay System (Beckman Coulter, Brea, CA, USA), respectively. PGE2 (PKGE004B; R&D Systems, Minneapolis, MN, USA) and PGF2α (ab133041; abcam, Cambridge, UK) levels were also measured for prostaglandin analysis.

### 2.7. Functional Fitness

The assessments of cardiovascular endurance, hand grip strength, explosive power, and core strength endurance were performed using 3 min step, hand dynamometer, horizontal jump, and bent-knee sit-up tests, respectively. The detailed procedures, devices, and manipulation were described in a previous study [22].

### 2.8. Sample Size Calculation

The required sample size was calculated using the repeated measures design equation of the G∗Power software (version 3.1.). The following parameters were used in the calculation: effect size (0.25), power (80%), type I error probability (0.05), five groups, and two repeated measurements [30]. It was determined that a total of 55 participants would be required to proceed with the exercise and probiotics intervention. Considering the approximately 10% estimated dropout rate, the goal was to recruit a total of 60 eligible participants.

### 2.9. Statistical Analysis

The sociodemographic and menstrual characteristics of the participants were analyzed using descriptive statistics and a chi-squared test. All dependent variables were assessed for normality, using Kolmogorov–Smirnov tests to perform the appropriate parametric or non-parametric analyses. Non-parametric methods, including the Wilcoxon signed-rank test, Pearson chi-squared test, and Kruskal–Wallis test, as well as parametric methods such as one-way analysis of variance (ANOVA) and paired *t*-tests, were employed to test for significant differences in dependent variables, both among and within the groups. SPSS version 22 (IBM, Armonk, NY, USA) was used for all statistical analyses. A probability of a type I error < 0.05 indicated statistical significance. The figures were prepared by GraphPad Prism Version 8.3.0 for Windows (GraphPad Software, Boston, MA, USA).

## 3. Results

### 3.1. Anthropometric and Menstrual Characteristics of Participants

No significant differences were found in the assessed anthropometric data, including age, height, weight, body mass index, heart rate, and both systolic and diastolic blood pressure, among the treatment groups (F(4, 60) = 0.788–1.895, *p* > 0.05) (Table 1). Similarly, there were no significant differences in menstrual factors such as age at menarche, menstrual cycle interval and duration, and menstrual flow across the study groups (F(4, 60) = 0.444–1.736, *p* > 0.05). However, significant differences were observed among the groups regarding the days of dysmenorrhea onset and the use of analgesics (F(4, 60) = 15.380–25.733, *p* < 0.05), with higher values reported in the primary dysmenorrhea (PD) groups compared to the control group. Moreover, there were no significant differences observed among groups in terms of physical activities and frequency (χ2 (8) = 4.61, *p* = 0.842).

### 3.2. Premenstrual Syndromes with HIIT and Probiotics Implementsation

The premenstrual syndromes were used to survey participants regarding symptoms prior to menstruation (Table 2). In the pre-test survey, the psychological and emotional (anger, anxiety, tearfulness, and depression) status; interest in work activity, home activity, and social activity; concentration; fatigue/lack of energy; overeating; hypersomnia; physical symptoms; interference with work productivity and relationships with colleagues; and social activity were ranked by perceived severity. These parameters and total scores displayed a significant increase in the PD group compared with the control group (participants were only divided into two groups, non-PD versus PD). After the 10-week program, the post-test survey showed that overall premenstrual symptoms exhibited significant improvement in the training groups (DysmenEx and DysmenExPro groups) compared with the Dysmen group; nonetheless, they remained significantly higher compared with those recorded in the control group. There was no significant difference in the total score of PSST observed between the Dysmen and DysmenPro groups; nevertheless, the overall premenstrual syndromes demonstrated significant improvement within the DysmenPro group. More specifically, emotions (anger, depression, and tearfulness) and fatigue were ameliorated within the DysmenPro group. In addition, symptoms (anger, anxiety, depressive mood, and social activities) were significantly ameliorated in the DysmenPro group compared with the Dysmen group. Regarding the effects of exercise alone (DysmenEx) or in combination with probiotic supplementation (DysmenExPro), the psychological and emotional (anger, anxiety, and depression) status, fatigue, hypersomnia, physical symptoms, interference with work productivity, and social activity were significantly mitigated within and among groups (in post-test analysis).

### 3.3. Effects of HIIT Based on MDQ and SF-MPQ

The MDQ and SF-MPQ were applied to evaluate symptoms associated with menstruation and to quantify the corresponding severity of pain (Table 3). In the pre-test survey, symptoms such as cramping, fatigue, breast pain or tenderness, general aches and pains, cold sweat, and skin blemishes, along with the total scores from the MDQ, were ranked according to perceived severity. A significant increase was observed in the PD group compared with the control group (participants were only divided into two groups). The results indicated that exercise training, alone or in combination with probiotics (DysmenEx and DysmenExPro groups, respectively), resulted in significant decreases in menstrual distress symptoms (cramp, fatigue, backache, swelling, painful or tender breast, general ache and pain) and total scores of MDQ in the post-test compared with the pre-test. In addition, there was no significant difference observed in the total scores of MDQ between the Dysmen and DysmenPro groups. However, there was a significant decrease in the symptoms of fatigue, general aches and pains, and total scores within the DysmenPro group. The overall menstrual distress symptoms were significantly ameliorated in the DysmenEx and DysmenExPro groups compared with the Dysmen and DysmenPro groups; nevertheless, they remained significantly higher than those recorded in the control group. In the survey of perceived pain intensity before intervention, the total scores from the sum of the participants obtained from the SF-MPQ and VAS surveys were significantly higher in the PD group than the control group (27.5 ± 5.9 versus 15.2 ± 0.6; 7.5 ± 1.1 versus 0.5 ± 0.7, respectively). Following the 10-week exercise intervention, perceived pain intensity showed a significant decrease in the DysmenEx and DysmenExPro groups compared with the Dysmen and DysmenPro groups. In conclusion, the total scores representing the overall severity of PD demonstrated that HIIT exercise training may have improved pain severity by approximately 30 mm on the VAS in the exercise groups (DysmenEx and DysmenExPro) groups compared with the Dysmen group.

### 3.4. Effects of HIIT and Probiotic Supplementation on Biochemical Variables and Hormones

There were no significant differences among groups in terms of biochemical markers (AST, ALT, BUN, creatinine, triglycerides, total cholesterol, LDH, and CPK) representing the indicated tissue function and physiological homeostasis (F(4, 60) = 0.052 to 1.081, *p* > 0.05) before intervention (pre-test) (Table 4). However, hsCRP demonstrated a significantly higher value (F(4, 60) = 13.107, *p* < 0.001) in the PD groups versus the control group. Moreover, significant differences in hormones (estradiol, prolactin, progesterone, and cortisol) were also observed among groups (F(4, 60) = 3.63–13.107, *p* < 0.05). The levels of estradiol and prolactin were significantly higher, whereas those of progesterone and cortisol were significantly lower, in the PD groups compared with the control group. No significant differences were observed in LH and FSH levels between the groups (F(4, 60) = 0.191, *p* = 0.940 and F(4, 60) = 0.379, *p* = 0.823, respectively).

After 10 weeks of HIIT and probiotic intervention (post-test), the levels of AST, ALT, BUN, creatinine, triglycerides, total cholesterol, LDH, CPK, LH, and FSH did not display significant differences among groups (F(4, 60) = 0.021–0.365, *p* > 0.05). Significant differences were observed among group levels of hsCRP (F(4, 60) = 9.786, *p* < 0.05), estradiol (F(4, 60) = 8.874, *p* < 0.05), prolactin (F(4, 60) = 6.366, *p* < 0.05), progesterone (F(4, 60) = 18.993, *p* < 0.05), and cortisol (F(4, 60) = 8.192, *p* < 0.05) (Table 4). More specifically, the DysmenEx and DysmenExPro groups (HIIT) exhibited significant increases in progesterone and cortisol levels, as well as decreases in hsCRP, estradiol, and prolactin levels, compared with the Dysmen and DysmenPro groups. However, those indices remained significantly higher (hsCRP and prolactin) or lower (progesterone and cortisol) in the training groups (DysmenEx and DysmenExPro groups) compared with the control group. By comparing the pre- and post-test assessments, we observed significant decreases in hsCRP and estradiol levels, in addition to increases in progesterone and cortisol levels, in the DysmenEx and DysmenExPro groups as a result of HIIT intervention. The effect of probiotics on dysmenorrhea (DysmenPro) also significantly decreased with the levels of estradiol within group.

### 3.5. Physical Fitness with HIIT and Probiotic Supplementation

Upper limb strength, explosive force, cardiovascular endurance, and core strength were assessed before and after the HIIT and probiotic intervention (Table 5). No significant differences were observed in grip strength, standing long jump distance, standing triple jump distance, 3 min step test, and bent-knee sit-up test measurements (F(4, 60) = 0.054–0.341, *p* > 0.05) among the three groups prior to the experimental intervention (pre-test). After the 10-week HIIT and probiotic intervention, grip strength, core strength (30 s), and endurance (60 s) did not show significant differences among or within groups. However, significant differences in physical fitness, namely cardiovascular endurance (F(4, 60) = 3.894, *p* < 0.05) and explosive force (F(4, 60) = 3.276, *p* < 0.05), were observed among groups. The 10-week HIIT resulted in significant improvements in explosive force and cardiovascular endurance in the DysmenEx and DysmenExPro groups compared with the control, Dysmen, and DysmenPro groups. Furthermore, the DysmenEx and DysmenExPro groups exhibited significant improvements in cardiovascular endurance and explosive force within groups, but the only probiotic supplementation group (DysmenPro group) demonstrated no significance between control and Dysmen groups or within the group itself.

### 3.6. Effects of HIIT on Prostaglandins Levels

**Figure 3:**
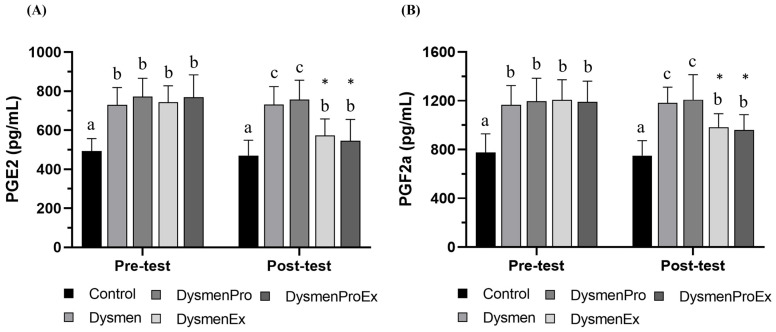
The effect of HIIT spinning exercises on prostaglandins levels. Blood samples were collected before and after experimental implementation to measure levels of PGE2 (**A**) and PGF2α (**B**). Data are presented as the mean ± SD. Different superscript letters (a, b, and c) indicate significant differences (*p* < 0.05), as evaluated by one-way ANOVA. * represents significant differences within groups.

In pre-test analyses, there were significant differences in the levels of PGE2 and PGE2α(F(4, 60) = 17.69, *p* < 0.0001 and F(4, 60) = 21.45, *p* < 0.0001, respectively) (Figure 3); the PGE2 and PGF2α levels in dysmenorrhea groups were significantly higher than those in the control group. Following the 10-week HIIT and probiotic supplementation, significant differences in PGE2 and PGF2α levels were observed between the groups (F(4, 60) = 12.95, *p* < 0.0001 and F(4, 60) = 19.43, *p* < 0.0001, respectively). Before the intervention, the levels of prostaglandins in the dysmenorrhea groups were significantly higher than those in the control group. Although the prostaglandins levels remained significantly higher in the DysmenEx and DysmenExPro groups (HIIT) compared with the control group, they were significantly decreased compared with those measured in the Dysmen and DysmenPro groups. Prostaglandins may be modulated as a result of the HIIT intervention, but not probiotic supplementation.

### 3.7. Correlation Between Hormones, hsCRP, and Prostaglandins Levels

Table 6 presents the results of independent correlation analyses among MDQ, SF-MPQ, prostaglandins, cortisol, progesterone, estradiol, hsCRP, and prolactin. Menstrual distress (MDQ) and pain severity (SF-MPQ) demonstrated a significant positive correlation with prostaglandins, hsCRP, and prolactin (r = 0.771–0.278, *p* < 0.05) and a significant negative correlation with cortisol and progesterone (r = −0.320–−0.522, *p* < 0.05). In addition, prostaglandins demonstrated a significant negative correlation with hormones (cortisol and progesterone) (r = −0.489–−0.401, *p* < 0.05; r = −0.593–−0.579, *p* < 0.05) and a significant positive correlation with estradiol, hsCRP, and prolactin (r = 0.315–0.549, *p* < 0.05). Regarding the correlation between hormones and inflammatory variables, progesterone showed a significant negative correlation with hsCRP (r = −0.627, *p* < 0.05), estradiol (r = −0.333, *p* < 0.05), and prolactin (r = −0.439, *p* < 0.05).

## 4. Discussion

PD is a condition characterized by physiological and psychological distress. Patients typically present with abdominal and lower back pain during the menstruation cycle; however, the pathological symptoms are not clinically acknowledged. Furthermore, a review study previously revealed that exercise with different modes, intensities, and durations could induce analgesia through central and peripheral mechanisms. The effects of exercise in females with chronic pain conditions should be investigated further [31]. In the present study, probiotic supplementation and exercise in the form of HIIT intervention were implemented to evaluate the effects on alleviation of PD. The results suggest that this exercise model could enhance physical attributes, such as cardiovascular endurance and explosive force. Additionally, premenstrual symptoms and menstrual distress were significantly alleviated following the 10-week probiotic supplementation and HIIT program. Moreover, the perceived pain severity in menstruation might be modulated with HIIT exercise intervention through the modulation of hormones and decreased inflammation.

The most common symptoms of PD included irritability, anxiety, abdominal discomfort, and heightened emotional sensitivity. Furthermore, an increased risk of moderate to severe PD-related pain intensity was associated with factors such as age, nulliparity, and the presence of PD since adolescence [32]. In Taiwan, the prevalence (70.7%) was also reported in individuals aged 22–48 years. The predictive factors included age <40 years, shift work, and marital status; attitudes towards menstruation showed significant association with these factors in the multiple logistic regression analysis [33]. In the present intervention study, we identified individuals with PD based on specific inclusion criteria (age, dysmenorrhea types, and pain severity). Similar menstrual distress syndromes, including physiological impacts, psychological distress, and interference in social activities, could be observed and these syndromes were further ameliorated after exercise and probiotic intervention. In addition, the premenstrual dysphoric disorder (the most severe form of premenstrual symptom) resulted in extreme mood shifts, with severe impacts on life quality, relationships, and hormone modulation (serotonin and progesterone). The interaction between peripheral inflammation and brain neurotransmitter systems may be a target for the development of new therapeutic strategies [34]. However, alternative nutritional approaches, including the use of probiotics and psychobiotics in the management of depression and anxiety, have been reported in recent years. The possible mechanisms underlying these approaches could be related to the synthesis of neurotransmitters (e.g., serotonin and gamma-aminobutyric acid), the modulation of inflammatory cytokines, or enhanced regulation of the hypothalamic–pituitary–adrenal axis and stress hormones; the optimal dosage and action mode should be investigated further in clinical trials [35]. In the present study, premenstrual syndromes (particularly the psychological and emotional status) could be significantly ameliorated through probiotic and exercise intervention. However, microbiota and metabolites should be further analyzed to identify the possible mechanisms underlying the alleviation of premenstrual syndrome and propose potential treatments for premenstrual dysphoric disorder.

The levels of phospholipid on the membrane are increased in response to an increase in progesterone levels after ovulation. Arachidonic acid is released with the progesterone downregulation of unfertilized condition and is metabolized by cyclooxygenase for prostaglandins (PGE2 and PGF2α) and leukotrienes. These eicosanoids further induce hypertonus and the vasoconstriction of the myometrium, ischemia-associated endometrial death, and an increase in the sensitivity of uterine pain nerve fibers. Prostaglandins demonstrated a direct correlation with menstrual pain intensity, cramps, and distress symptoms in PD; pharmacological or alternative modalities were mainly used for pain alleviation [36]. However, the risk of venous thromboembolism was positively associated with NSAID treatment widely used for PD amelioration. In women of reproductive age, a higher rate of venous thromboembolism events was observed in those using NSAIDs combined with high levels of hormonal contraception compared with those using low levels of hormonal contraception [37]. In previous studies on treatments for PD, the levels of prostaglandins fluctuated according to the sampling time points during the menstrual cycle; however, higher levels of prostaglandins were seen in women with PD versus healthy women. In a previous study on the effect of traditional Chinese medicine supplementation on PD, the PGE2 levels were higher than those of PGF2α on the second day of the menstrual cycle and supplementation significantly ameliorated the pain intensity through prostaglandin regulation [38]. Before menstruation sampling, the levels of PGF2α were significantly higher than those of PGE2. These prostaglandins, as well as inflammatory and oxidative markers (Hs-CRP and malondialdehyde), could also be modulated with chlorella supplementation to improve pain and systemic symptoms [39]. In the present study, we found that HIIT exercise could effectively modulate prostaglandins for the management of dysmenorrhea, possibly with fewer health risks compared to pharmaceutical use, but that probiotic treatments should be developed and investigated further. However, the detailed fluctuations in prostaglandins warrant further validation during the menstrual cycle.

Progesterone levels fluctuate over the lifespan of females, as evidenced by increased concentrations at puberty, cyclical changes during the menstrual cycle, a steady rise during pregnancy, and rare expression in menopause. Progesterone plays an important role in reproduction and anti-inflammation by binding to the glucocorticoid receptor to inhibit the mitogen-activated protein kinase (MAPK) pathway and downstream cyclooxygenase-2 and interleukin-1β (IL-1β) expression [40]. In serial serum assessment during the menstrual cycle, menstruation-related symptoms (abdominal cramps, and gastrointestinal and pain symptoms) were positively correlated with C-C motif chemokine ligand 5 (CCL5) and insulin-like growth factor 1 (IGF-1); however, they were negatively correlated with progesterone [41]. In addition, an association study between hormones and PD demonstrated that higher estradiol levels were associated with higher frequency of dysmenorrhea and degree of pain [42]. However, hormones can show different modulations after exercise intervention in different physiological statuses. A previous study also revealed that exercises can reduce hormone levels (estradiol and progesterone) in premenopausal women with high-risk breast cancer to achieve incremental benefits in surgical or pharmacologic interventions [43]. Contrary to our results, significantly increased estrogen and decreased progesterone levels could be observed immediately after acute maximal aerobic exercise in health female athletes [44]. Another study focused on the acute HIIT exercise with excess post-exercise oxygen consumption and the data showed the significant modulation of estradiol and progesterone in the luteal phase [45]. Thus, factors including sampling time, the exercise program, and physiological status could also contribute to the regulation of and fluctuations in related hormones. Moreover, the present long-term HIIT exercise may induce effective adaptation and hormone homeostasis with clinical significance.

A study also indicated that the occurrence of both premenstrual syndrome and primary dysmenorrhea was associated with an altered prooxidant–antioxidant balance, higher levels of hsCRP, and an increased neutrophil-to-lymphocyte ratio [46]. The nutritional strategy, oeoylethanolamide supplementation, could alleviate dysmenorrhea through the modulation of inflammation (malondialdehyde, CRP, and TNF-α) [47]. Cortisol levels exhibited a decreasing trend from the follicular to the luteal phase of the menstrual cycle. Mean cortisol was significantly lower in women with dysmenorrhea and was negatively correlated with the duration of symptoms [48]. Additionally, an increase in prolactin concentration has been suggested as a potential risk factor for the pathogenesis of adenomyosis. The use of a prolactin inhibitor may significantly improve menstrual bleeding, reduce pain, and enhance quality of life [49]. In the present study, the modulation of hsCRP, cortisol, and prolactin levels in patients with dysmenorrhea was significantly improved after the completion of a 10-week HIIT exercise, but not as a result of probiotic supplementation.

Based on a previous spinning bike HIIT protocol [22], we modified the all-out phase to out-of-saddle sprinting with higher resistance for higher exertion. Multiple physiological adaptations of HIIT that improve exercise capacity and metabolic health in different populations have been reported [50]. A meta-review also addressed the effects of HIIT on the improvement of cardiorespiratory fitness, some inflammatory markers, muscle structure, anxiety, and depression [51]. Physiological adaptation varies according to the exercise implementation in relation to population and exercise program. In postmenopausal women, elevation in monocyte chemoattractant protein-1 (MCP-1), IL-6, and IL-10 levels, as well as lipid peroxidation (thiobarbituric acid reactive substances) and advanced oxidation protein products, was observed immediately after acute HIIT exercise. However, the 4-week HIIT intervention could significantly regulate cytokines (IL-6, IL-10, and IL-1α) to exert anti-inflammatory effects, with limited changes in oxidative stress markers [52]. In the present study, the exercise frequency of lifestyle did not show a significant difference among groups, but it did not show the beneficial effects on Dysmen group possibly due to the insufficient intensity implementation and arrangement. Mitochondrial biogenesis related to cellular stress and the resultant metabolic signals highly depend on exercise intensity with respect to physiological adaptations and interval training, clearly exerting a potent stimulus for physiological remodeling in humans [53]. Therefore, physiological regulation, such as hormones and inflammation, and menstrual distress syndromes related to dysmenorrhea may be modulated by the HIIT intervention.

Regarding the effects of HIIT on physical fitness, the neuromuscular status, anaerobic power, and acute heart rate recovery were significantly improved in athletes after HIIT implementation [54]. In the present study, the 3 min step test was used to measure the levels of aerobic (cardiovascular) fitness based on the heart rate recovery at the same stepping pace intensity. In a previous report, the 3 min step test was utilized to predict the VO_2_max in healthy adults. Nonetheless, the validity and reliability of the equation should be further identified in a different population [55]. With regard to the effects of HIIT intervention on explosive power, a systematic review indicated that HIIT has the potential to enhance power while positively influencing critical variables associated with both aerobic and anaerobic endurance, including VO_2_max, running. and repetitive sprint performance, jumping ability, and hitting speed during gameplay [56].

In the present study, physical fitness, cardiovascular endurance, and explosive power were significantly improved following the implementation of the 10-week HIIT program; nevertheless, probiotic supplementation did not result in significant improvement.

The evidence supporting the use of probiotics in the management of conditions, such as depression, anxiety, and stress-related disorders, was examined through systematic analysis, observational studies, and meta-analyses. The cognitive and psychological functions could be modulated by changes in the gut microbiota through the microbiota–gut–brain axis; hence, it was concluded that probiotics may provide novel, personalized treatment options for the treatment of depression and anxiety [57]. Following multi-strain probiotic supplementation, serotonin was increased after 6 weeks of supplementation; this effect was accompanied by a lower state of anxiety, reduced trait anxiety, and a lower Leiden Index of Depression Sensitivity-Revised (hopeless, aggression, rumination, and total score) [58]. Intervention with *L. plantarum* JYLP-326 could be considered as an effective supplementary strategy for the amelioration of anxiety, depression, and insomnia in test-anxious students, working via microbiota restoration and metabolite modulation [59]. Therefore, the probiotics that confer mental health benefits are termed psychobiotics and the certain strains of probiotics, such as Bifidobacterium and Lactobacillus, allow the modulation of the gut microbial system in order to achieve benefits at the psychological, immune, hormonal, and mental levels through metabolites or neurochemicals [60]. In the present survey of premenstrual symptoms, significant improvements were observed in anxiety and depression after exercise intervention and were also seen in the probiotic supplementation-only group (DysmenPro) compared with the dysmenorrhea group (Dysmen). The microbiota and metabolites may be affected by exercise training and probiotic supplementation in terms of mood mitigation in PD. However, further investigation in possible mechanisms underlying the corresponding psychological benefits and physiological improvement in cases of dysmenorrhea is warranted.

## 5. Conclusions

The evidence also revealed high heterogeneity regarding the type of exercise, the use of a combination of exercises or one exercise alone, and the volume of interventions on pain mitigation of PD [61]. Therefore, we implemented a targeted exercise program, specifically adapted to a high-intensity interval training (HIIT) model, and probiotic supplementation to enhance physical fitness, psychological resilience, and physiological adaptation. However, there are several variable factors, including exercise types adapted for HIIT models, age, population, and nutritional supplementation (probiotics), that may influence the effects of this on dysmenorrhea improvement. From the perspective of health promotion, the arrangement and monitoring of exercise intensity may play a critical role in the amelioration of PD through multiple physiological adaptations rather than frequency. Considering different types of dysmenorrhea, diverse populations, variations in exercise prescriptions, and treatment duration could help to elucidate the underlying mechanisms of probiotics and exercise intervention. Further investigation into the multiple physiological axes, microbiota, and metabolites involved in this process is warranted. Omics methodologies, such as metagenomics, metabolomics, and proteomics, could serve as valuable tools for revealing the mechanistic regulation of treatments in the context of dysmenorrhea. The strategy discussed in the present study (i.e., exercise and/or probiotics intervention) may be useful in promoting women’s health and improving their quality of life. Furthermore, it may be employed as a preventive method against gynecological disorders in the adolescent population.

## Figures and Tables

**Table 1:** Anthropometric and menstrual characteristics of the participants.

Characteristic	Control	Dysmen	DysmenPro	DysmenEx	DysmenExPro
Age (years)	24.4 ± 6.5	24.5 ± 4.8	21.8 ± 3.9	21.9 ± 2.5	24.9 ± 7.4
Height (cm)	158 ± 4.5	161 ± 4.0	161 ± 6.2	159 ± 4.6	162 ± 4.9
Weight (kg)	54.2 ± 6.2	56.0 ± 7.0	51.7 ± 5.9	54.1 ± 6.4	56.0 ± 8.7
BMI (kg/m^2^)	21.6 ± 2.3	21.7 ± 2.4	20.0 ± 2.1	21.3 ± 2.4	21.3 ± 2.9
Systolic blood pressure (mmHg)	114 ± 13	107 ± 9.6	108 ± 9.6	115 ± 9.7	113 ± 6.5
Diastolic blood pressure (mmHg)	76 ± 6.1	75 ± 7.2	71 ± 7.9	74 ± 8.0	74 ± 6.2
Resting heart rate (bpm)	80 ± 11.8	81 ± 6.5	85 ± 10.6	79 ± 12.6	80 ± 10.9
Menarche age (years)	12.0 ± 1.2	12.1 ± 1.0	12.3 ± 1.0	12.5 ± 1.2	12.3 ± 0.9
Interval of menstrual cycle (days)	28.1 ± 1.3	30.0 ± 1.7	30.0 ± 1.8	29.0 ± 1.7	29.9 ± 3.5
Duration of menstrual cycle (days)	4.9 ± 1.2	5.2 ± 1.1	5.5 ± 0.9	5.4 ± 1.1	5.3 ± 1.1
Menstrual flow ^+^	1.8 ± 0.6	2.2 ± 0.7	2.1 ± 0.5	1.7 ± 0.6	2.0 ± 0.6
Days with dysmenorrhea (during menses)	0.1 ± 0.3 ^a^	2.9 ± 1.1 ^b^	2.9 ± 1.6 ^b^	2.8 ± 0.6 ^b^	3.0 ± 1.1 ^b^
Painkiller medication (times)	0.2 ± 0.6 ^a^	3.8 ± 1.7 ^b^	3.6 ± 1.1 ^b^	3.9 ± 1.2 ^b^	4.1 ± 1.7 ^b^
Exercise frequency					
None	6	5	8	6	5
≤3 times/week	4	7	7	7	6
≥4 times/week	0	1	0	1	2

Data are presented as the mean ± SD. ^+^ indicates perceived menstrual flow, rated as light, medium, or heavy (score 1 to 3). The superscripts a and b indicate significant differences (*p* < 0.05) between groups.

**Table 2:** Mean score and intensity of premenstrual syndrome before and after the HIIT exercise and probiotic intervention.

PSST Component	Control Group		Dysmen Group		DysmenPro Group		DysmenEx Group		DysmenExPro Group	
	Rank Mean		Rank Mean		Rank Mean		Rank Mean		Rank Mean	
Symptom	Pre	After	Pre	After	Pre	After	Pre	After	Pre	After
Anger/irritability	1.4 ± 0.5 ^a^	1.4 ± 0.5 ^a^	2.3 ± 0.7 ^ab^	2.4 ± 0.8 ^b^	2.4 ± 0.7 ^b^	1.8 ± 0.6 ^a^*	2.1 ± 0.8 ^ab^	1.6 ± 0.5 ^a^	2.4 ± 0.8 ^b^	1.7 ± 0.6 ^a^*
Anxiety/tension	1.1 ± 0.3 ^a^	1.2 ± 0.4 ^a^	1.7 ± 0.9 ^ab^	2.2 ± 0.8 ^b^	1.9 ± 0.6 ^b^	1.7 ± 0.6 ^a^	1.9 ± 0.9 ^ab^	1.6 ± 0.6 ^a^	2.0 ± 0.8 ^b^	1.7 ± 0.6 ^a^
Tearfulness	1.2 ± 0.4 ^a^	1.3 ± 0.6	1.5 ± 0.7 ^a^	1.4 ± 0.7	2.3 ± 0.9 ^ab^	1.8 ± 0.6 *	1.9 ± 1.0 ^a^	1.6 ± 0.7	1.8 ± 1.1 ^a^	1.7 ± 0.7
Depressed mood	1.3 ± 0.5 ^a^	1.3 ± 0.5 ^a^	2.5 ± 0.7 ^b^	2.5 ± 0.7 ^c^	2.5 ± 0.6 ^b^	1.8 ± 0.7 ^ab^*	2.4 ± 0.9 ^b^	1.8 ± 0.7 ^ab^*	2.4 ± 1.1 ^b^	1.8 ± 0.6 ^b^*
Decreased interest in work activities	1.3 ± 0.5 ^a^	1.4 ± 0.5 ^a^	2.2 ± 0.8 ^ab^	2.2 ± 0.9	2.4 ± 1.1 ^b^	2.2 ± 0.9	2.1 ± 0.9 ^ab^	1.6 ± 0.5	2.1 ± 0.6 ^ab^	1.7 ± 0.5
Decreased interest in home activities	1.1 ± 0.3	1.3 ± 0.5	1.5 ± 0.5	1.8 ± 0.6	2.0 ± 0.9	1.5 ± 0.6	1.6 ± 0.9	1.7 ± 0.6	1.8 ± 0.7	1.5 ± 0.7
Decreased interest in social activities	1.2 ± 0.4	1.3 ± 0.5 ^a^	2.1 ± 0.8	2.3 ± 0.9 ^b^	2.1 ± 1.0	2.1 ± 0.7 ^b^	1.9 ± 0.8	1.9 ± 0.9 ^ab^	2.1 ± 0.8	1.8 ± 0.6 ^ab^
Difficulty concentrating	1.0 ± 0 ^a^	1.1 ± 0.3 ^a^	1.8 ± 0.7 ^ab^	1.9 ± 0.7 ^b^	2.1 ± 0.8 ^b^	1.7 ± 0.7 ^b^	1.8 ± 0.9 ^ab^	1.7 ± 0.7 ^b^	1.9 ± 1.0 ^ab^	1.8 ± 0.6 ^b^
Fatigue/lack of energy	1.5 ± 0.5 ^a^	1.4 ± 0.5 ^a^	2.9 ± 0.9 ^b^	2.9 ± 0.9 ^c^	3.0 ± 0.8 ^b^	2.5 ± 0.7 ^bc^*	2.6 ± 0.9 ^ab^	2.1 ± 0.6 ^b^*	2.9 ± 1.1 ^b^	2.3 ± 0.5 ^b^
Overeating/food cravings	1.4 ± 0.5	1.5 ± 0.7	2.2 ± 1.1	2.1 ± 0.9	2.3 ± 1.1	2.1 ± 1.1	2.2 ± 0.8	2.1 ± 0.8	2.3 ± 0.9	2.0 ± 1.0
Insomnia	1.1 ± 0.3	1.0 ± 0	1.6 ± 0.9	1.6 ± 0.9	1.3 ± 0.6	1.3 ± 0.6	1.2 ± 0.6	1.4 ± 0.6	1.5 ± 0.5	1.5 ± 0.5
Hypersomnia	1.3 ± 0.5 ^a^	1.4 ± 0.5 ^a^	2.4 ± 1.1 ^b^	2.6 ± 1.0 ^c^	2.6 ± 0.9 ^b^	2.3 ± 1.0 ^bc^	2.3 ± 0.9 ^ab^	1.8 ± 1.0 ^ab^	2.4 ± 0.7 ^b^	1.8 ± 0.6 ^ab^*
Feeling overwhelmed/out of control	1.2 ± 0.4	1.0 ± 0 ^a^	1.3 ± 0.6	1.3 ± 0.6 ^ab^	1.9 ± 0.9	1.6 ± 0.7 ^b^	1.5 ± 0.8	1.3 ± 0.5 ^ab^	1.4 ± 0.5	1.3 ± 0.5 ^ab^
Physical symptoms ^+^	1.4 ± 0.5 ^a^	1.4 ± 0.5 ^a^	2.4 ± 0.7 ^b^	2.5 ± 0.9 ^b^	2.6 ± 0.7 ^b^	2.4 ± 0.6 ^b^	2.4 ± 0.6 ^b^	1.9 ± 0.4 ^a^*	2.5 ± 0.6 ^b^	1.8 ± 0.4 ^a^*
Interference with activities										
Work productivity/efficiency	1.1 ± 0.3 ^a^	1.1 ± 0.3 ^a^	1.9 ± 0.6 ^b^	1.8 ± 0.5 ^b^	2.0 ± 0.7 ^b^	1.5 ± 0.6 ^ab^	2.0 ± 0.8 ^b^	1.4 ± 0.5 ^ab^*	1.9 ± 0.8 ^ab^	1.5 ± 0.5 ^ab^*
Colleagues/classmate	1.0 ± 0	1.0 ± 0	1.4 ± 0.7	1.5 ± 0.7	1.3 ± 0.5	1.2 ± 0.4	1.4 ± 0.8	1.4 ± 0.5	1.3 ± 0.5	1.2 ± 0.4
Family relationships	1.0 ± 0	1.0 ± 0	1.5 ± 0.8	1.3 ± 0.5	1.4 ± 0.6	1.4 ± 0.7	1.2 ± 0.8	1.3 ± 0.5	1.4 ± 0.5	1.3 ± 0.5
Social activities	1.4 ± 0.5 ^a^	1.0 ± 0 ^a^	2.0 ± 0.8 ^b^	2.1 ± 0.9 ^b^	1.9 ± 0.6 ^b^	1.4 ± 0.7 ^a^	1.8 ± 0.8 ^ab^	1.5 ± 0.5 ^a^	1.8 ± 0.7 ^ab^	1.4 ± 0.5 ^a^*
Home responsibilities	1.0 ± 0	1.0 ± 0	1.2 ± 0.6	1.2 ± 0.4	1.2 ± 0.4	1.3 ± 0.6	1.1 ± 0.3	1.1 ± 0.4	1.2 ± 0.6	1.0 ± 0
**Total scores**	22.7 ± 3.4 ^a^	23.1 ± 3.8 ^a^	36.7 ± 9.8 ^b^	37.6 ± 7.6 ^c^	39.3 ± 8.5 ^b^	33.7 ± 8.0 ^bc^*	35.5 ± 9.9 ^b^	30.7 ± 6.7 ^b^*	37.2 ± 10 ^b^	30.8 ± 4.7 ^b^*

^+^ indicates physical symptoms of premenstrual syndrome, including breast tenderness, headaches, joint/muscular pain, bloating, and weight gain. Data are presented as the mean ± SD. The superscript (a, b, and c)indicate significant differences (*p* < 0.05) between groups pre- or post-testing. * indicates significant differences within groups.

**Table 3:** The mean score and intensity results in terms of Menstrual Distress and Short-Form McGill Pain Questionnaire scores before and after the HIIT exercise and probiotic intervention.

Distress Component	Control Group		Dysmen Group		DysmenPro Group		DysmenEx Group		DysmenExPro Group	
	Rank Mean		Rank Mean		Rank Mean		Rank Mean		Rank Mean	
Item	Pre	Post	Pre	Post	Pre	Post	Pre	Post	Pre	Post
Cramp	1.0 ± 0 ^a^	1.0 ± 0 ^a^	2.8 ± 0.9 ^b^	2.7 ± 0.6 ^c^	2.5 ± 0.9 ^b^	2.1 ± 0.6 ^b^	2.7 ± 0.7 ^b^	2.1 ± 0.7 ^b^*	2.8 ± 0.8 ^b^	2.0 ± 0.7 ^b^*
Fatigue	1.7 ± 0.5 ^a^	1.5 ± 0.5 ^a^	2.7 ± 0.7 ^b^	2.7 ± 0.6 ^d^	2.9 ± 0.7 ^b^	2.5 ± 0.5 ^cd^*	2.7 ± 0.6 ^b^	2.1 ± 0.5 ^bc^*	2.8 ± 0.7 ^b^	2.0 ± 0.6 ^b^*
Backache	1.7 ± 0.7	1.5 ± 0.5 ^a^	2.2 ± 1.9	2.2 ± 0.8 ^b^	2.3 ± 0.9	2.0 ± 0.9 ^ab^	2.4 ± 0.6	1.8 ± 0.6 ^ab^*	1.9 ± 0.7	1.6 ± 0.5 ^ab^
Swelling (chest/abdomen)	1.8 ± 0.6	1.4 ± 0.5 ^a^	2.1 ± 0.8	2.1 ± 0.5 ^bc^	2.3 ± 0.7	2.5 ± 0.9 ^c^	2.4 ± 0.6	1.6 ± 0.5 ^ab^*	2.3 ± 0.6	2.1 ± 0.5 ^bc^
Painful or tender breast	1.2 ± 0.4 ^a^	1.3 ± 0.5 ^a^	2.1 ± 0.8 ^b^	2.0 ± 0.6 ^b^	2.4 ± 0.8 ^b^	2.2 ± 1.0 ^b^	2.4 ± 0.8 ^b^	1.6 ± 0.5 ^ab^*	2.3 ± 0.8 ^b^	1.8 ± 0.8 ^ab^
General ache and pains	1.4 ± 0.5 ^a^	1.3 ± 0.5 ^a^	2.5 ± 1.1 ^b^	2.6 ± 0.9 ^b^	2.3 ± 0.8 ^b^	1.6 ± 0.6 ^a^*	2.4 ± 0.8 ^b^	1.6 ± 0.6 ^a^*	2.1 ± 0.9 ^ab^	1.5 ± 0.6 ^a^*
Dizziness	1.1 ± 0.3	1.1 ± 0.3	1.7 ± 0.9	1.4 ± 0.5	1.5 ± 0.9	1.6 ± 0.9	1.5 ± 0.9	1.3 ± 0.5	1.5 ± 0.7	1.2 ± 0.4
Cold sweat	1.0 ± 0	1.0 ± 0	1.5 ± 0.9	1.1 ± 0.3	1.3 ± 0.5	1.2 ± 0.4	1.4 ± 0.9	1.2 ± 0.4	1.5 ± 0.7	1.2 ± 0.4
Headache	1.3 ± 0.5 ^a^	1.4 ± 0.7	2.2 ± 1.1 ^b^	1.9 ± 1.0	1.6 ± 0.9 ^ab^	1.3 ± 0.6	1.5 ± 0.9 ^ab^	1.5 ± 0.8	1.8 ± 0.9 ^ab^	1.5 ± 0.5
Nausea/vomiting	1.0 ± 0	1.0 ± 0	1.5 ± 0.8	1.3 ± 0.6	1.2 ± 0.4	1.2 ± 0.4	1.4 ± 0.6	1.3 ± 0.5	1.4 ± 0.7	1.2 ± 0.4
Hot flashes	1.3 ± 0.5	1.3 ± 0.5 ^ab^	1.5 ± 0.8	1.3 ± 0.5 ^ab^	1.6 ± 0.7	1.6 ± 0.7 ^a^	1.3 ± 0.5	1.1 ± 0.3 ^b^	1.7 ± 0.9	1.3 ± 0.5 ^ab^*
Muscle stiffness	1.0 ± 0	1.0 ± 0	1.2 ± 0.6	1.2 ± 0.4	1.3 ± 0.6	1.3 ± 0.6	1.1 ± 0.3	1.1 ± 0.3	1.2 ± 0.4	1.1 ± 0.3
Swelling legs	1.2 ± 0.4 ^a^	1.2 ± 0.4 ^a^	1.9 ± 0.9 ^ab^	1.4 ± 0.7 ^ab^	2.1 ± 1.1 ^b^	1.8 ± 0.8 ^b^	1.3 ± 0.5 ^a^	1.3 ± 0.6 ^a^	1.4 ± 0.9 ^ab^	1.2 ± 0.4 ^a^
Heart pounding	1.0 ± 0 ^a^	1.0 ± 0	1.5 ± 0.8 ^b^	1.3 ± 0.6	1.3 ± 0.6 ^ab^	1.3 ± 0.4	1.1 ± 0.4 ^ab^	1.3 ± 0.4	1.2 ± 0.4 ^ab^	1.1 ± 0.3
Skin blemish or disorder	1.2 ± 0.4 ^a^	1.5 ± 0.7 ^a^	2.3 ± 1.0 ^b^	2.2 ± 1.0 ^b^	2.5 ± 1.1 ^b^	2.2 ± 0.9 ^b^	1.8 ± 0.7 ^ab^	1.8 ± 0.4 ^ab^	2.0 ± 0.7 ^b^	1.7 ± 0.6 ^ab^
Numbness	1.0 ± 0	1.0 ± 0	1.2 ± 0.4	1.2 ± 0.4	1.1 ± 0.4	1.1 ± 0.3	1.0 ± 0	1.0 ± 0	1.0 ± 0	1.1 ± 0.3
**Total score**	19.9 ± 1.9 ^a^	19.5 ± 2.8 ^a^	30.9 ± 8.4 ^b^	28.4 ± 4.8 ^c^	30.2 ± 5.7 ^b^	27.5 ± 5.2 ^c^*	28.5 ± 3.8 ^b^	23.5 ± 4.2 ^b^*	28.8 ± 5.4 ^b^	23.5 ± 4.9 ^b^*
Short-Form McGill Pain Questionnaire (total score)	15.2 ± 0.6 ^a^	15.3 ± 0.9 ^a^	27.4 ± 5.7 ^b^	27.3 ± 8.1 ^c^	27.5 ± 4.8 ^b^	26.3 ± 4.1 ^c^	27.5 ± 6.7 ^b^	21.1 ± 4.3 ^b^*	27.5 ± 7.0 ^b^	21.6 ± 6.5 ^b^*
Visual analog scale of pain	0.5 ± 0.7 ^a^	0.4 ± 0.7 ^a^	7.6 ± 0.8 ^b^	7.5 ± 1.0 ^c^	7.4 ± 0.9 ^b^	6.9 ± 1.1 ^c^	7.5 ± 1.4 ^b^	4.4 ± 1.8 ^b^*	7.5 ± 1.1 ^b^	4.3 ± 1.7 ^b^*

Data are presented as the mean ± SD. The superscript (a, b, c, and d) indicate significant differences (*p* < 0.05) between groups pre- or post-testing. * indicates significant differences within groups.

**Table 4:** Effects of HIIT spinning exercise and probiotic intervention on biochemical variables and hormones.

	Control Group		Dysmen Group		DysmenPro Group		DysmenEx Group		DysmenExPro Group	
	Pre	Post	Pre	Post	Pre	Post	Pre	Post	Pre	Post
AST (U/L)	14.3 ± 3.3	14.3 ± 2.1	15.2 ± 2.6	14.3 ± 4.3	15.6 ± 2.9	14.5 ± 3.3	14.4 ± 2.6	14.1 ± 3.2	15.1 ± 2.4	14.4 ± 3.7
ALT (U/L)	6.3 ± 1.9	6.1 ± 1.4	7.3 ± 1.9	6.6 ± 2.7	6.9 ± 1.7	6.8 ± 2.8	6.9 ± 2.3	6.8 ± 2.7	7.6 ± 1.8	6.8 ± 3.4
BUN (mg/dL)	11.2 ± 1.8	11.1 ± 2.8	11.1 ± 3.0	11.2 ± 1.7	11.5 ± 3.7	11.6 ± 1.6	11.4 ± 2.9	11.9 ± 2.7	11.4 ± 1.9	11.2 ± 2.9
CREA (mg/dL)	0.68 ± 0.14	0.65 ± 0.13	0.67 ± 0.09	0.64 ± 0.08	0.63 ± 0.09	0.62 ± 0.11	0.64 ± 0.09	0.64 ± 0.11	0.59 ± 0.13	0.63 ± 0.11
TG (mg/dL)	68.4 ± 17	68.0 ± 15	69.1 ± 17	67.3 ± 15	72.7 ± 14	70.7 ± 14	65.3 ± 18	64.9 ± 19	67.0 ± 13	68.7 ± 14
CHOL (mg/dL)	172 ± 24	172 ± 34	175 ± 29	170 ± 24	173 ± 26	171 ± 22	168 ± 29	169 ± 20	170 ± 26	171 ± 26
LDH (U/L)	114 ± 15	117 ± 14	119 ± 16	120 ± 17	116 ± 22	115 ± 17	113 ± 13	115 ± 11	117 ± 21	120 ± 16
CPK (U/L)	65 ± 29	66 ± 22	64 ± 29	63 ± 25	65 ± 25	65 ± 20	66 ± 32	67 ± 27	62 ± 22	66 ± 25
HsCRP (mg/dL)	0.044 ± 0.04 ^a^	0.045 ± 0.04 ^a^	0.170 ± 0.05 ^b^	0.186 ± 0.08 ^c^	0.171 ± 0.06 ^b^	0.144 ± 0.07 ^bc^	0.182 ± 0.05 ^b^	0.108 ± 0.05 ^b^*	0.177 ± 0.04 ^b^	0.114 ± 0.03 ^b^*
Estradiol (pg/mL)	147 ± 26 ^a^	144 ± 21 ^a^	187 ± 25 ^b^	188 ± 26 ^b^	183 ± 27 ^b^	166 ± 29 ^ab^*	181 ± 27 ^b^	137 ± 25 ^a^*	180 ± 31 ^b^	136 ± 31 ^a^*
FSH (mIU/mL)	3.2 ± 1.2	3.2 ± 1.1	3.1 ± 1.3	3.4 ± 1.6	3.0 ± 1.2	3.0 ± 1.3	3.1 ± 1.1	3.3 ± 0.9	3.5 ± 1.3	3.7 ± 2.0
LH (mIU/mL)	5.1 ± 1.8	4.9 ± 1.7	5.0 ± 1.5	5.3 ± 1.5	4.9 ± 2.1	5.0 ± 2.0	5.5 ± 2.6	6.2 ± 2.4	5.0 ± 2.4	5.4 ± 2.3
Prolactin (ng/mL)	12.7 ± 5.0	15.2 ± 3.6 ^a^	20.7 ± 6.2	25.3 ± 8.7 ^c^	21.2 ± 6.8	25.4 ± 5.2 ^c^	22.1 ± 7.8	20.0 ± 5.5 ^b^	21.3 ± 4.7	20.4 ± 4.0 ^b^
Progesterone (ng/mL)	12.3 ± 4.4 ^b^	13.1 ± 4.0 ^c^	4.8 ± 3.5 ^a^	4.8 ± 2.3 ^a^	4.4 ± 2.9 ^a^	5.7 ± 2.3 ^a^	4.1 ± 2.7 ^a^	10.4 ± 2.9 ^b^*	4.0 ± 2.8 ^a^	10.8 ± 2.7 ^b^*
Cortisol (μg/dL)	11.3 ± 2.7 ^b^	13.1 ± 3.5 ^c^	6.4 ± 2.4 ^a^	6.7 ± 3.8 ^a^	6.5 ± 2.3 ^a^	7.5 ± 2.6 ^a^	7.0 ± 2.6 ^a^	10.5 ± 2.6 ^b^*	7.2 ± 2.5 ^a^	10.6 ± 3.2 ^b^*

Data are presented as the mean ± SD. The superscript a, b, and c indicate significant differences (*p* < 0.05) between groups pre- or post-testing. * indicates significant differences within groups.

**Table 5:** Effects of HIIT spinning exercise and probiotic supplementation intervention on physical fitness.

	Control Group		Dysmen Group		DysmenPro Group		DysmenEx Group		DysmenExPro Group	
	Pre	Post	Pre	Post	Pre	Post	Pre	Post	Pre	Post
Grip strength (kg)	26.7 ± 5.5	25.4 ± 4.6	26.4 ± 2.5	25.0 ± 3.7	24.9 ± 4.2	24.1 ± 4.6	25.3 ± 4.6	26.1 ± 4.3	25.6 ± 5.4	25.3 ± 4.6
Standing long jump (cm)	154 ± 22	154 ± 20 ^a^	155 ± 16	154 ± 13 ^a^	157 ± 21	154 ± 18 ^a^	158 ± 29	170 ± 24 *^b^	160 ± 19	171 ± 16 *^b^
Standing triple jumps (cm)	434 ± 79	424 ± 80 ^a^	437 ± 58	420 ± 60 ^a^	431 ± 55	428 ± 57 ^a^	452 ± 68	483 ± 71 *^b^	431 ± 33	478 ± 33 *^b^
3 min step test	52 ± 5.6	52 ± 4.2 ^a^	51 ± 4.9	52 ± 5.0 ^a^	52 ± 5.6	52 ± 5.9 ^a^	52 ± 7.8	59 ± 9.7 *^b^	52 ± 5.5	58 ± 7.4 *^b^
Bent-knee sit-up										
30 s	17.8 ± 5.0	15.8 ± 2.9	17.7 ± 6.2	16.9 ± 4.4	17.6 ± 5.3	18.1 ± 2.3	18.4 ± 5.5	19.6 ± 6.1	17.5 ± 3.7	17.2 ± 6.1
60 s	31.1 ± 8.6	31.7 ± 7.8	30.4 ± 11	29.0 ± 11	32.7 ± 10	31.9 ± 10	33.1 ± 10	32.3 ± 10	33.0 ± 8.4	32.3 ± 11

Data are presented as the mean ± SD. The superscript a and b indicate significant differences (*p* < 0.05) between groups pre- or post-testing. * indicates significant differences within groups.

**Table 6:** Correlation of menstrual distress, pain intensity, hormones, and inflammatory variables with HIIT exercise and probiotic intervention in women with dysmenorrhea.

	MDQ	SF-MPQ	PGE2	PGF2α	Cortisol	Progesterone	Estradiol	HsCRP	Prolactin
MDQ	1	0.771 **	0.379 **	0.447 **	−0.320 *	−0.522 **	0.235	0.408 **	0.278 *
SF-MPQ	0.771 **	1	0.508 **	0.588 **	−0.364 **	−0.493 **	0.174	0.317 *	0.378 **
PGE2	0.379 **	0.508 **	1	0.497 **	−0.489 **	−0.593 **	0.469 **	0.467 **	0.495 **
PGF2α	0.447 **	0.588 **	0.497 **	1	−0.401 **	−0.578 **	0.395 **	0.549 **	0.315 *
Cortisol	−0.320 *	−0.364 **	−0.489 **	−0.401 **	1	0.328 *	−0.322 *	−0.373 *	−0.256 *
Progesterone	−0.522 **	−0.493 **	−0.593 **	−0.579 **	0.328 *	1	−0.333 **	−0.627 **	−0.439 **
Estradiol	0.235	0.174	0.469 **	0.395 **	−0.322 *	−0.333 **	1	0.278 *	0.321 **
HsCRP	0.408 **	0.317 *	0.467 **	0.549 **	−0.373 *	−0.627 **	0.278 *	1	0.180
Prolactin	0.278 *	0.378 **	0.495 **	0.315 *	−0.256 *	−0.439 **	0.321 *	0.180	1

* and ** indicate significant differences between variables (*p* < 0.05 and *p* < 0.01, respectively).

## Data Availability

The original contributions presented in this study are included in the article. Further inquiries can be directed to the corresponding author.
